# Quantitative analysis of idiopathic epiretinal membrane traction: an updated version of the relaxation index

**DOI:** 10.3389/fopht.2025.1528766

**Published:** 2025-05-22

**Authors:** Davide Allegrini, Raffaele Raimondi, Giovanni Montesano, Marco Caruso, Giovanna Lionetti, Adriano Carnevali, Tania Sorrentino, Vincenzo Scorcia, Mario R. Romano

**Affiliations:** ^1^ Eye Center, Humanitas Gavazzeni-Castelli, Bergamo, Italy; ^2^ Department of Biomedical Sciences, Humanitas University, Pieve Emanuele, Italy; ^3^ Optometry and Visual Sciences, City St George’s, University of London, London, United Kingdom; ^4^ NIHR Biomedical Research Centre, Moorfields Eye Hospital NHS Foundation Trust and UCL, Institute of Ophthalmology, London, United Kingdom; ^5^ PolitoBIOMed Lab—Biomedical Engineering Lab and Department of Electronics and Telecommunications, Politecnico di Torino, Torino, Italy; ^6^ Università degli Studi Magna Graecia di Catanzaro, AOU Renato Dulbecco, Dipartimento di Scienze Mediche e Chirurgiche, Catanzaro, Italy

**Keywords:** relaxation index, epiretinal membrane, tangential traction, vitreoretinal surgery, infrared image

## Abstract

**Purpose:**

The aim of this work was to track tangential traction of idiopathic epiretinal membrane from an initial assessment to the immediate post-operative phase using an enhanced version of the relaxation index (RI).

**Methods:**

A retrospective analysis was conducted on 9 patients who underwent peeling surgery for idiopathic, symptomatic, and progressive epiretinal membrane. The RI assesses the displacement of vascular crossings in time from a fixed point, which is the retinal pigmented epithelium. This updated iteration integrates infrared images paired with OCT scans instead of OCTA.

**Results:**

The study encompassed three timepoints: T1 (initial appointment), T2 (1 week pre-surgery), and Post (1 month post-surgery). T1 was 12± 9 months prior to surgery. A statistically significant difference (p<0.001) in RI was observed across all three timepoints; however, there was no significant correlation between RI and visual acuity (p>0.05).

**Conclusion:**

The RI emerges as a comprehensive and direct parameter for objectively assessing and monitoring tangential traction in three dimensions across an extensive area of the posterior pole. Further streamlining of the process is necessary to integrate this feature into clinical practice effectively.

## 1 Introduction

The epiretinal membrane (ERM) is a fibrocellular overgrowth at the interface between the vitreous and the retina, situated above the inner limiting membrane (ILM) (1). The incidence of this condition rises with age and has been reported to reach up to 28.9% in the United States population (2). This fibrotic process is driven by the accumulation of collagen and the transformation of retinal Müller cells, retinal pigmented epithelium (RPE) cells, and hyalocytes into myofibroblasts (3, 4). Ultimately, this transformation leads to contraction and subsequent puckering of the macular area. As a result, this membrane exerts traction on the retina, ultimately causing metamorphopsias and reduced visual acuity (5). Currently, surgical peeling is the only therapeutic option (6).

The evolution of these changes are not currently directly quantified by physicians with an objective measure, but is rather assessed by clinicians through visual inspection of series of images of the retina (7). Consequently, surgical timing remains uncertain and there is a wide disparity in approaches among surgeons (7). Quantitative assessments of retinal changes have become more widespread with the introduction of Optical Coherence Tomography (OCT) imaging, which allows precise measurements of retinal morphology (8).

Because the development of ERMs is often accompanied by changes in retinal thickness, this parameter is often used as a surrogate measure of change (9). However, this alone does not directly quantify many important features of ERMs. In a previous publication, we introduced the relaxation index (RI), a measure to quantify the three-dimensional shift in position of vascular landmarks before and after ERM peeling surgery (10), showing that this measurement was linked to post-operative best corrected visual acuity (**BC**VA) recovery. In this manuscript, we want to instead focus on using this measurement to track the natural evolution of ERMs in OCT scans, measuring the progression of traction from a baseline assessment up to the immediate post-operation period.

## 2 Materials and methods

### 2.1 Sample description

The electronic medical records of the Ophthalmology Department of Humanitas Gavazzeni-Castelli Hospital of Bergamo and at the Germaneto University Hospital were queried to identify all patients 18 years or older affected by idiopathic, symptomatic and progressive epiretinal membrane (ERM) that underwent peeling surgery between March and August 2022 (n = 67). Search was performed on 5th of November 2022 on de-identified data. The progression was defined as a decrease of visual function, the presence of concomitant metamorphopsia and increased central macular thickness secondary to epiretinal traction. Exclusion criteria were: myopia greater than 6 diopters (n = 13), a history of ocular surgeries including cataract surgery (n = 20), macular edema secondary to vascular and tractional diseases (n = 4), previous uveitis (n=2), diabetic retinopathy (n = 8), age-related macular degeneration (n = 2), complete follow up data not available (n = 9).Therefore, we retrospectively analyzed 9 eyes from 9 patients who underwent peeling of the ERM and the ILM to treat visually significant ERM. Written informed consent before surgery was obtained from all subjects, this study conformed to the Declaration of Helsinki and ethics approval was approved by the Ethics Committee of Humanitas Gavazzeni Hospital with the protocol number 42/20 GAV.

Surgeries were performed by two different surgeons (M.R.R.) and (A.C.). All patients were examined at the first visit (T1), one week before surgery (T2) and one month after surgery (T-post). At all visits, best corrected visual acuity (BCVA) was measured in decimals and SD-OCT was performed using the follow-up mode.

### 2.2 Relaxation index

As already described, RI measures in millimetres (mm), the shift in position of a vascular crossing from a reference point, defined as the vertical projection of that same crossing onto the RPE, to the new position of the reference points obtained in the follow-up visits (10). Assuming that the outer retina is anchored to the RPE so that no horizontal sliding of the retina is possible, the stretch of the tissue over time can be measured (11).

RI was measured comparing ten reference points manually selected (vessel crossing) for each patient at the first visit to that one week before surgery (T1-T2), comparing reference points at the first visit to that one month after surgery (T1-Tpost) and finally comparing reference points at the visit one week before surgery to that at the visit one month after surgery (T2-Tpost). The RI used for the analysis is the average of the shifts of all the crossings Therefore RI can be measured at every timepoint to measure an increase in tangential traction. Indeed, this shift represents a measure of the pre- and post-operative movement of the retina due to traction and its relaxation after surgery.

### 2.3 Images acquisition

Spectralis SD-OCT (Heidelberg Engineering, Germany) was used to acquire infrared reflectance (IR) fundus picture and OCT scans of the posterior pole at all time points with a field of view of 30°, using the follow-up mode. This employs a fundus tracking technology to ensure that OCT acquisitions are performed at the same location on the retina.

IR is performed by a light source with a wavelength of 815 nm that allows comfortable imaging and high-resolution visualization at the level of EPR.

OCT B-scans are performed by a light source of 880 nm (range of infrared) with an A-scan rate that goes from 40 to 85 kHz and an axial resolution of 3.9 μm/pixel.

We use as reference image an infrared (IR) fundus picture paired with the OCT B-scan since the acquisition was paired, the length of each arrow that we measured on IR was equal to the width of the B-mode image when both are expressed in μm .

Axial length was measured with a LS 900 TC optical low-coherence reflectometry biometer (Haag-Streit Diagnostics, Koeniz, Switzerland).

### 2.4 Image processing

The following processing was applied for each image at each time-point of each subject to each of the ten markers manually identified on the IR image by two scientists (R.R.) and (G.M).

The first step consisted of the grayscale conversion of the image acquired by the Spectralis SD-OCT. Then, it was necessary to separate the two images (IR and B-scan) to apply the relevant analysis to the specific image of interest. This is achieved by computing the horizontal gradient to identify the separation boundary.

#### 2.4.1 Analysis performed on the B-Mode image

**Figure 1:**
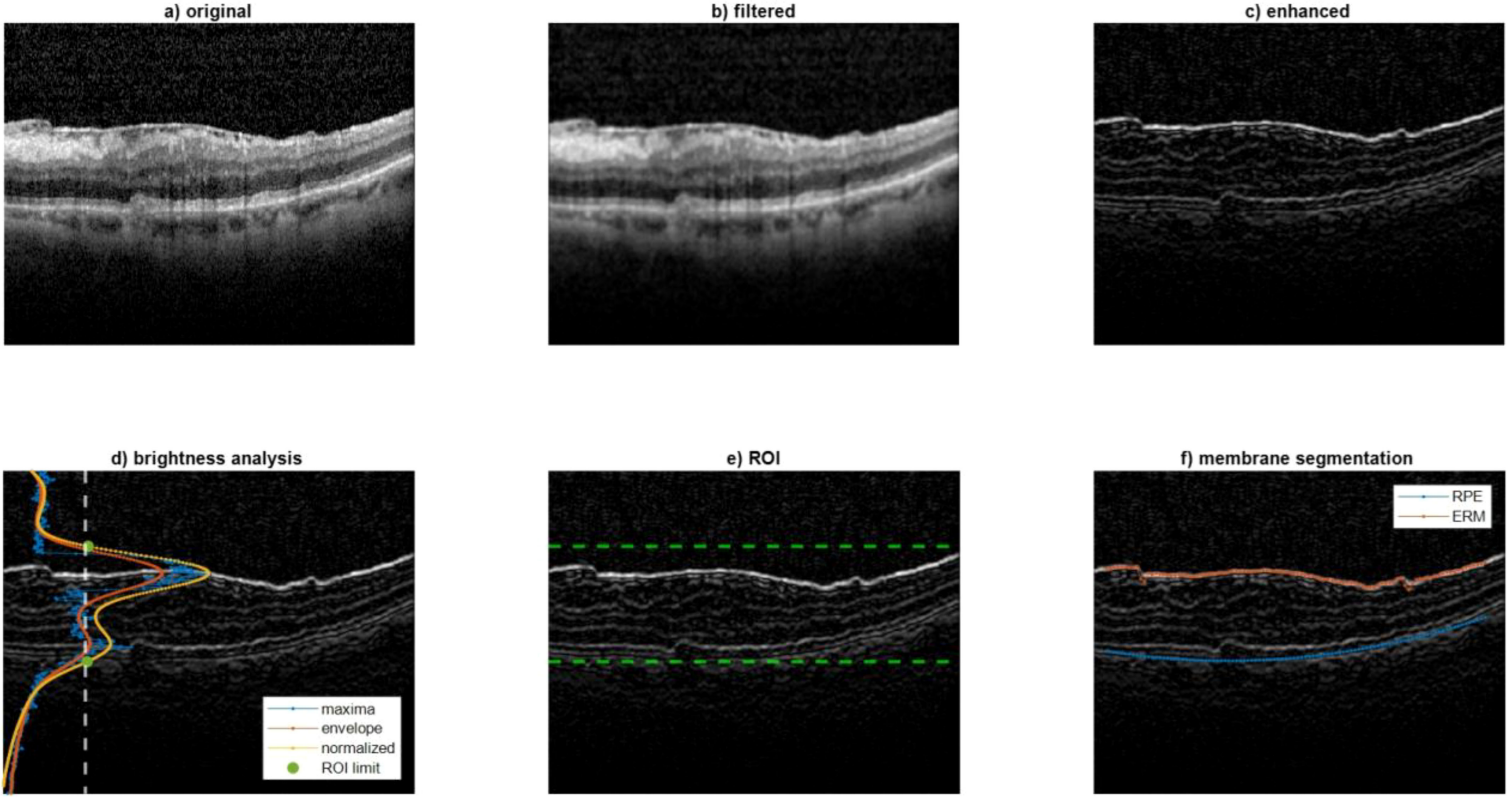
Analysis performed on the B-Mode image. This figure illustrates the image processing steps used to segment the epiretinal membrane (ERM) and retinal pigmented epithelium (RPE) layers on OCT B-Mode images. **(a)** Original B-Mode image; **(b)** smoothed image using a low-pass filter; **(c)** enhanced image with edge sharpening; **(d)** envelope of maximum intensity across rows used to define the region of interest (ROI); **(e)** detection of ROI boundaries (green dots); **(f)** final segmentation result with ERM and RPE highlighted in red and blue, respectively. These segmentations are used to track retinal displacement over time.

The following processing was aimed at segmenting the ERM and RPE on the B-Mode image (**Figure 1a**). Firstly, the B-Mode image was smoothed using a low-pass filter (average kernel 5x5), **Figure 1b**. After that, one region of interest (ROI) was defined to contain only the portion of image included between the ERM and RPE. It was hypothesized that this ROI could be identified by an increase of local brightness. To this end, some processing steps were needed. At first the image was enhanced by summing its high pass filtered image to sharpen the edges (**Figure 1c**). Then, the envelope of the maximum of each row was computed and then normalized between 0 and 1 (**Figure 1d**). The ROI was defined between the rows where the normalized envelope was higher than a threshold set to 0.4 (the limits of the ROI were detected as in **Figure 1e** and represented as green points superimposed to the normalized envelope). For each column of the ROI, the ERM and RPE were identified at the two intensity peaks closest to the ROI limits. To make this identification more robust to noise, the points whose vertical coordinates deviated from the average neighbor positions higher than a threshold were discarded. Finally, the ERM and RPE points were interpolated over 100 points using a spline and a 2^nd^ order polynomial, respectively. **Figure 1f** represents an example of segmentation of ERM and RPE which are represented in red and blue, respectively.

#### 2.4.2 Analysis performed on the IR image

**Figure 2:**
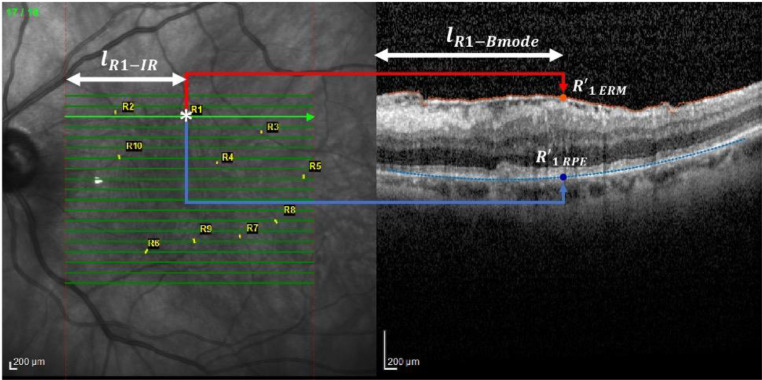
Analysis performed on the IR image. This figure shows the method for locating vessel crossings on infrared (IR) fundus images and correlating them with OCT B-scan locations. The green arrows mark the horizontal correspondence between IR and B-scan. The user identifies vessel crossing points (example: R1), and their positions are used to track displacement over time. The distances from the start of the IR scan to the vessel and its projection onto ERM and RPE are measured to calculate the Relaxation Index. *, scan point on IR image.

A color filter was employed to quantify the length (in terms of number of columns) corresponding to the horizontal green arrows (**Figure 2**, left).

Then, an operator, thanks to a zoomed view, clicked on the points corresponding to each marker whose coordinates were than saved.

The horizontal coordinate of the marker was used to compute the distance with respect to the begging of the IR scan ( $l_{R-IR}$ , as reported on the left panel of **Figure 2** for marker R1 as an example). This distance was used to locate the projection of the marker (whose horizontal coordinate with respect to the B-mode scan is represented by $l_{R-Bmode}$ , as reported on the right panel of **Figure 2** for marker R1) on the ERM and RPE, respectively, namely $R_{ERM}^{'}$ and $R_{RPE}^{'}$ . This was possible by exploiting the knowledge that the length of each arrow was equal to the width of the B-mode image when both are expressed in μm .

#### 2.4.3 Displacement computation

**Figure 3:**
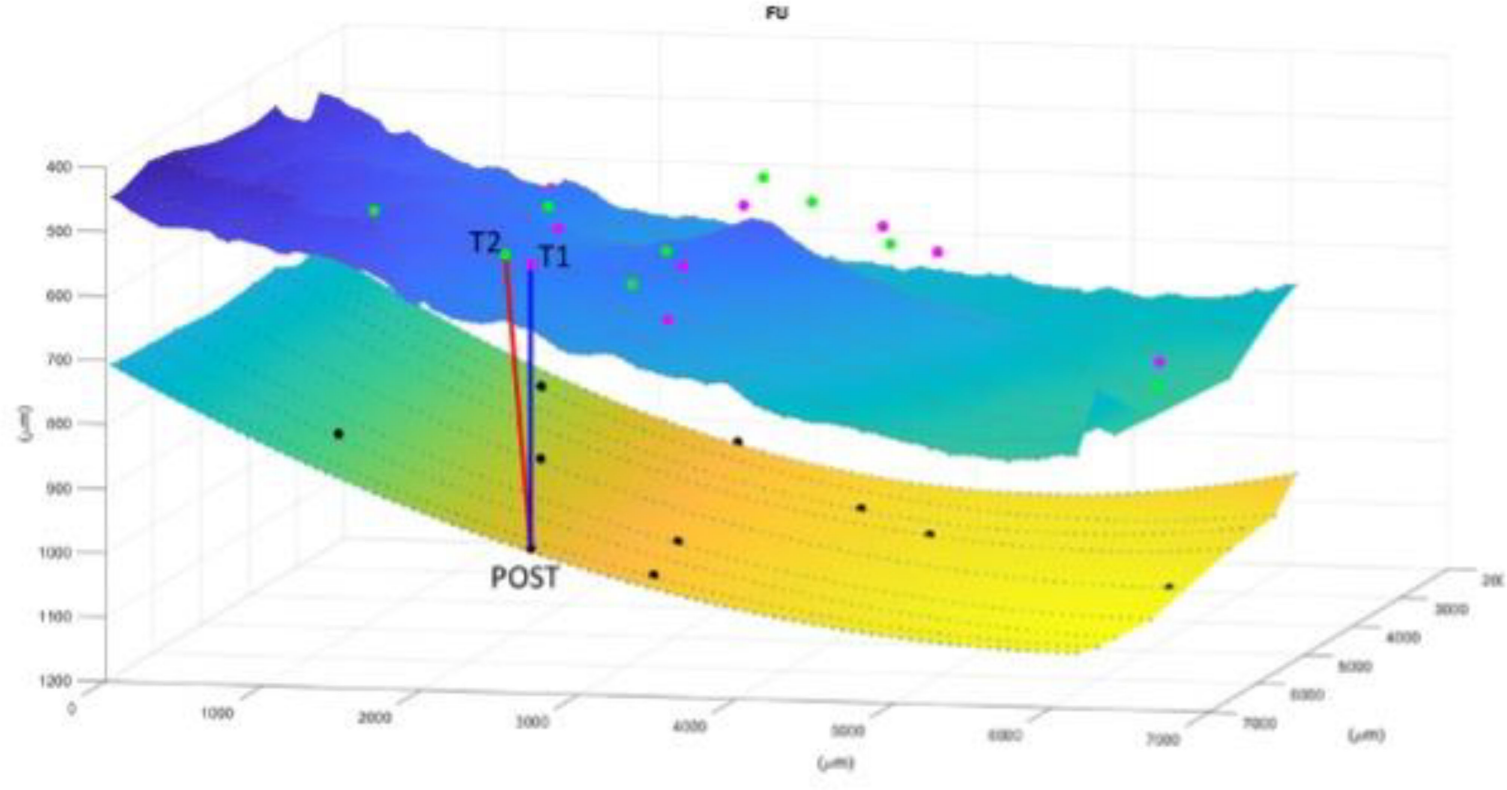
Image representation of displacement computation for each marker and for each of the three time points. The superior layer is the ERM and the inferior is the RPE. T1 (first appointment), T2 (1 week before surgery), POST (1 month after surgery). The Relaxation Index(RI) is the distance between the point at the RPE that vertically corresponds to the vessel crossing at POST and the same vessels crossing before surgery. In this case it is evident how the tangential traction is increased from the T1 to T2 and then released to almost the initial stage after surgery.

After having repeated this processing for each marker and for each of the three time points, the displacement is computed as in **Figure 3**.

### 2.5 Statistical analysis

The pointwise change in RI was quantified using a linear mixed effect model, fitted with the package lme4 for R (R Foundation for Statistical Computing, Vienna). For this analysis, the time points were used as discrete fixed effects, to account for the non-linear behavior over time. Random intercept terms were added for the eye and the marker, to account for correlations between successive measurements within the same eye and on the same vessel crossing. These random effect terms were nested. The package lsmeans for R was used to perform *post-hoc* pair-wise comparisons between time-points (Bonferroni-Holm correction for multiple testing).

The change in BCVA was calculated in a similar fashion, but with only a random intercept term for the eye. A model using the RI as a continuous fixed effect was used to test correlations between BCVA and RI.

## 3 Results

The study analyzed 9 eyes from 9 patients (average age: 81 ± 4 years). The mean time between the first recorded visit (T1) and surgery was 12 ± 9 months. At T2 (preoperative), the average BCVA was 0.54 ± 0.18.

### 3.1 Relaxation index

There was a statistically significant change in the RI between all three evaluated timepoints (T1, T2, and Post), with p < 0.001 for all pairwise comparisons.

Between T1 and T2, the RI increased in all patients, indicating a progression of tangential traction over time. This was observed as a gradual outward shift in the location of vascular landmarks. The mean RI showed consistent growth during this interval.

Following surgery, between T2 and Post the RI significantly decreased in every patient, suggesting a release of retinal traction. Interestingly, the RI at the Post timepoint approached the levels seen at T1, implying that surgery effectively reversed much of the progressive traction observed prior to the intervention.

**Figure 4:**
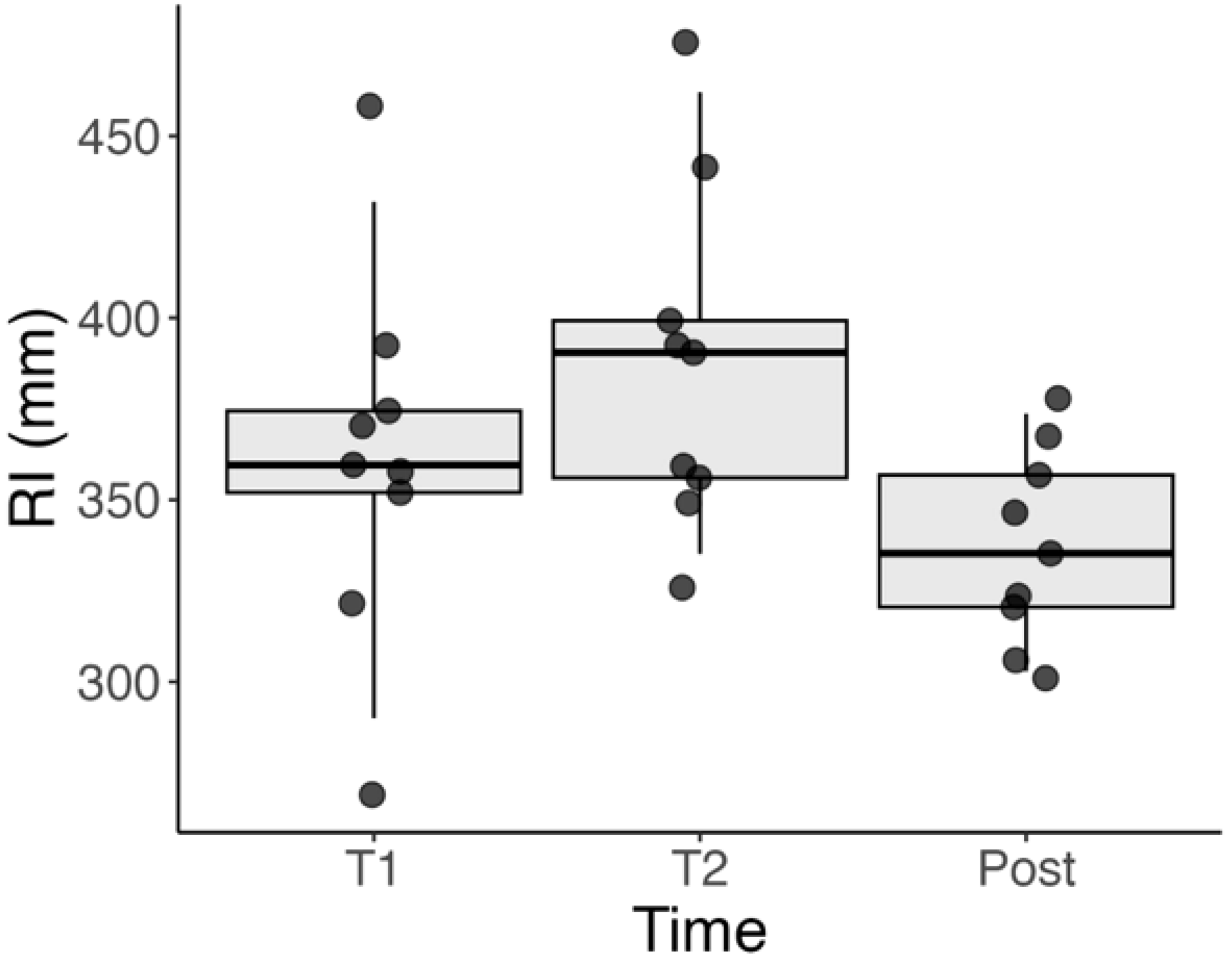
Boxplot showing the distribution of RI values at T1 (initial visit), T2 (1 week before surgery), and Post (1 month after surgery). A significant increase in RI is observed between T1 and T2, indicating progression of tangential retinal traction. A subsequent decrease from T2 to Post reflects the release of traction following surgical peeling. Statistical significance was observed between all three timepoints (p < 0.001).

These trends were visually consistent across all individual patients, as seen in **Supplementary Figure 1**, and are summarized in **Figure 4**.

### 3.2 BCVA

**Figure 5:**
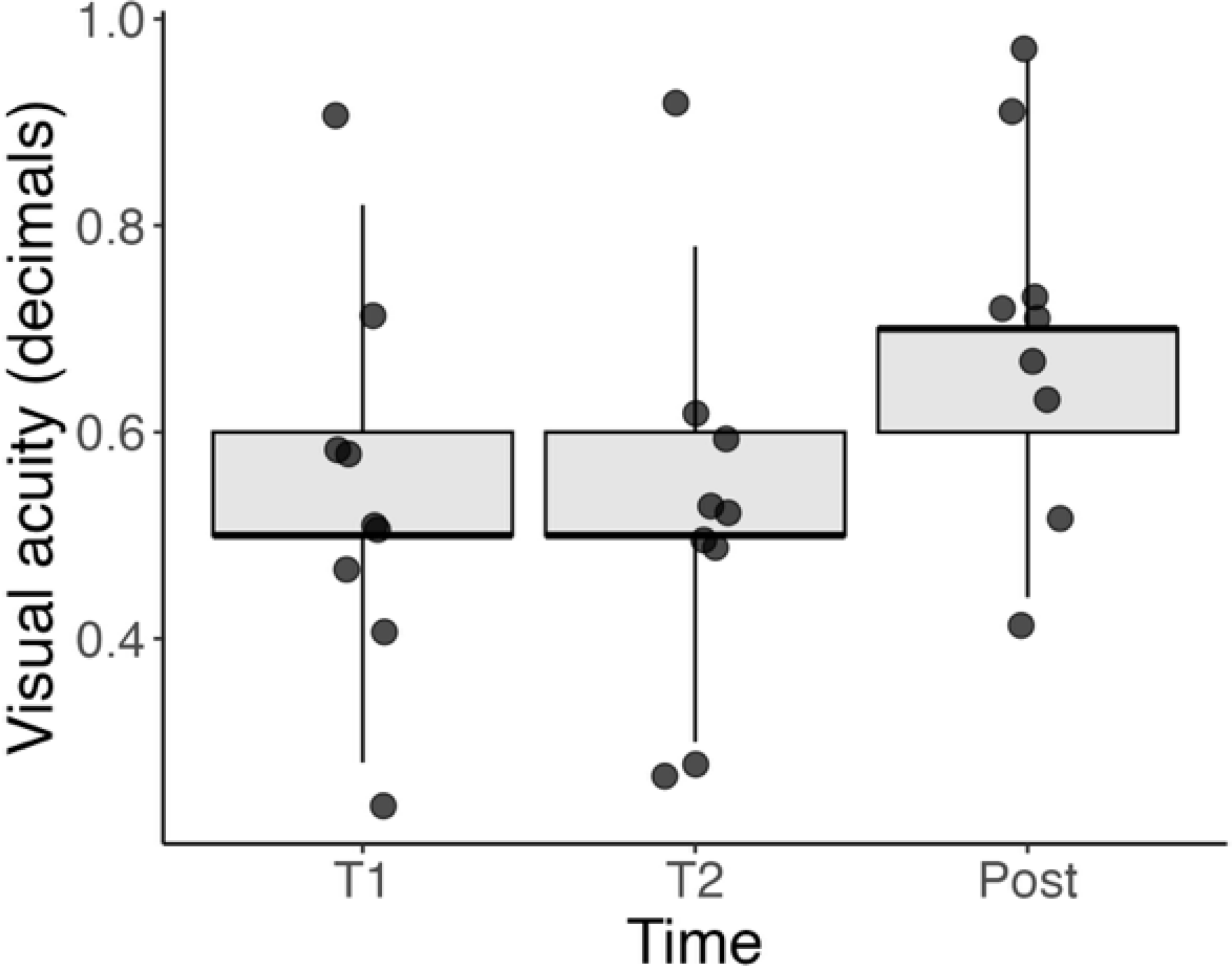
Boxplot of BCVA (in decimal values) at three timepoints: T1 (initial visit), T2 (1 week before surgery), and Post (1 month after surgery). While individual patients showed improvement after surgery, no statistically significant correlation was found between RI and BCVA either pre-operatively or overall. These data highlight the complex relationship between anatomical and functional recovery.

There was no significant correlation between the RI and VA either in the entire series (p = 0.225) or when isolating the pre-operative measurements (p = 0.604). **Figure 5** shows the BCVA over the three time-points. Like for the RI, there was a significant difference between the post-operative BCVA and the two pre-operative time points (both p < 0.001). However, there was no significant difference between the two pre-operative time points (p = 0.423).

Plotted BCVA of single patients at the different timepoints is included in **Supplementary Figure 2**.

## 4 Discussion

We present an updated method of processing images to obtain the previously proposed RI (10). This method has demonstrated effectiveness in monitoring traction from the initial follow-up, through the pre-operative phase, to the release of traction after surgery. Unlike the previous method, we no longer rely on OCTA images for tracking vessel crossings. Instead, these are manually marked on the infrared (IR) image, significantly expediting the imaging processing speed. It’s worth noting that in this version, we did not prioritize assessing functional outcomes. There was a statistically significant improvement in the BCVA after surgery. However, we did not identify a statistically significant correlation between the RI and BCVA), which could be attributed to the limited size of the cohort analyzed.

In our previous study, we examined patients one week before surgery, and at 1, 3, and 6 months post-surgery (10). We observed that the RI stabilized at its final state one month after the operation. Therefore, in this analysis, we concentrated on the progression of traction in the preoperative period from the initial visit to one month after surgery. We observed a statistically significant difference in the RI across all the timepoints. We studied the progression of tangential traction from the first appointment, to one week before surgery and at one month after surgery. Even if this is a small cohort there is a clear trend that shows how the traction increases and is then released after surgery. This method is consistent in allowing quantitative analysis of tangential traction which is a key feature in epiretinal membrane pathophysiology. Objective measurement of tangential traction should in our opinion guide clinical decisions.

Despite recent advancements in OCT analysis tools, the classification of ERM remains predominantly qualitative (7). Various classification systems have been proposed by different authors. Hwang et al. introduced a system that relies on foveal characteristics, validated with multifocal electroretinography (mERG), to delineate functional distinctions between stages. Konidaris et al. suggested a categorization based on vitreous state and structure, but this classification lacks validation, rendering its clinical utility uncertain (12). Stevenson et al. advocated considering central foveal thickness and inner segment ellipsoid band integrity as critical morphological parameters (13). Additionally, Govetto et al. established a four-stage classification system, centered on the presence of the foveal pit and the integrity of outer and inner retinal layers (7).

Another study underscored the significance of traction as a pivotal prognostic factor, particularly focusing on intra-retinal changes and measuring traction depth (14). Quantifying intraretinal morphologic changes remains a challenge. Furthermore, several investigations have explored the relationship between the morphology of retinal layers and BCVA, revealing that the morphology of the inner segment/outer segment (IS/OS) layer can serve as a predictive indicator for functional outcomes. Finally, there is an increasing interest at evaluation the effect of traction on ectopic inner foveal layers and its correlation with functional outcomes. Zhang Z. et al. found that stereoscopic foveal deviations significantly correlated with the baseline and postoperative visual acuity (15).

The introduction of the RI is aimed at directing scientific attention towards the macular region as a three-dimensional entity. The effect of the ERM traction on the retina indeed, as the alteration of the foveal morphology and retinal layers disorganization, can be caused both by the vertical traction and horizontal tangential forces.

While a three-dimensional evaluation is achievable with en-Face OCT, the study of tractional forces is uniquely possible with the RI. In our view, the RI represents a powerful index that now requires augmentation with extensive clinical data to fully demonstrate its clinical utility.

The current version of the RI incorporates several noteworthy updates, as previously discussed in the methods section. However, there are certain limitations that currently constrain the applicability of this method. Primarily, the manual marking of vascular crossings remains a significant bottleneck, in our view. Addressing this limitation is crucial to enable the widespread application of this method, as it is currently time-consuming. In this regard, our forthcoming work is dedicated to leveraging deep learning algorithms to automate vessel detection. Additionally, we applied stringent inclusion criteria and only assessed this on a small cohort of patients, which limits the robustness of our findings. Visual acuity was recorded in decimals and not in LogMAR. Lastly, we did not find any correlation with the functional method, which restricts the practical implications of this data to a pure study of the tractional forces without immediate clinical relevance.

## 5 Conclusion

The Relaxation Index emerges as a comprehensive and straightforward parameter for objectively measuring and tracking ERM traction in three dimensions across a wide area of the posterior pole, both pre- and post-surgery. Further studies involving larger cohorts are necessary, and potential integration with built-in software may establish it as a critical parameter for making objective decisions regarding surgical timing.

## Data Availability

The raw data supporting the conclusions of this article will be made available by the authors, without undue reservation.

## Supplementary material

Supplementary Figure 1 Line plots of RI values for each of the 9 patients across the three timepoints (T1, T2, Post). Each line represents one patient’s progression, showing a consistent pattern of increased RI prior to surgery and a decrease post-operatively, indicating consistent resolution of tangential traction.

Supplementary Figure 2 Line plots of BCVA values for each of the 9 patients across the three timepoints (T1, T2, Post). Some patients demonstrated improvement following surgery, though individual variability was notable. The plots provide insight into the functional outcomes alongside anatomical changes.
