# Supplementary material for: Quantitative analysis of idiopathic epiretinal membrane traction: an updated version of the relaxation index
Source: Front Ophthalmol (Lausanne). 2025 May 22;5:1528766. doi: 10.3389/fopht.2025.1528766 (PMC12137094; doi:10.3389/fopht.2025.1528766)
Supplement: Supplementary Figure 1 — Line plots of RI values for each of the 9 patients across the three timepoints (T1, T2, Post). Each line represents one patient’s progression, showing a consistent pattern of increased RI prior to surgery and a decrease post-operatively, indicating consistent resolution of tangential traction. [file DataSheet1.docx]

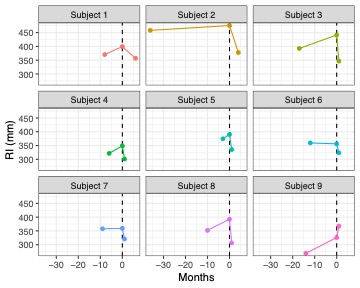


Supplementary figure 1.

Line plots of RI values for each of the 9 patients across the three timepoints (T1, T2, Post). Each line represents one patient’s progression, showing a consistent pattern of increased RI prior to surgery and a decrease post-operatively, indicating consistent resolution of tangential traction.


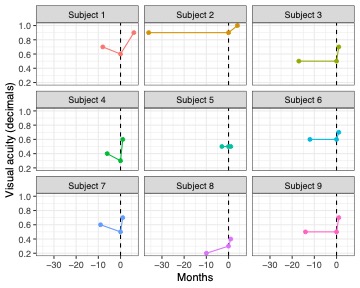


Supplementary figure 2.

Line plots of BCVA values for each of the 9 patients across the three timepoints (T1, T2, Post). Some patients demonstrated improvement following surgery, though individual variability was notable. The plots provide insight into the functional outcomes alongside anatomical changes.
